# Cannabinoid Effects on Experimental Colorectal Cancer Models Reduce Aberrant Crypt Foci (ACF) and Tumor Volume: A Systematic Review

**DOI:** 10.1155/2020/2371527

**Published:** 2020-07-20

**Authors:** Eduardo Orrego-González, Luisa Londoño-Tobón, José Ardila-González, Diego Polania-Tovar, Ana Valencia-Cárdenas, Alberto Velez-Van Meerbeke

**Affiliations:** ^1^Research Group, Neurosciences (NEUROS), School of Medicine and Health Sciences, Universidad Del Rosario, Bogotá, Colombia; ^2^Hospital Universitario Mayor (MEDERI), Bogotá, Colombia

## Abstract

**Objective:**

Colorectal cancer represents a heavy burden for health systems worldwide, being the third most common cancer worldwide. Despite the breakthroughs in medicine, current chemotherapeutic options continue to have important side effects and may not be effective in preventing disease progression. Cannabinoids might be substances with possible therapeutic potential for cancer because they can attenuate the side effects of chemotherapy and have antiproliferative and antimetastatic effects. We aim to determine, through a systematic review of experimental studies performed on animal CRC models, if cannabinoids can reduce the formation of preneoplastic lesions (aberrant crypt foci), number, and volume of neoplastic lesions.

**Materials and Methods:**

A systematic, qualitative review of the literature was conducted in accordance with Preferred Reporting Items for Systematic Reviews and Meta-Analyses (PRISMA) guidelines. PubMed, Embase, and Scopus databases were searched. We use the following Medical Subject Headings (MESH) terms in PubMed: “colorectal neoplasms,” “colonic neoplasms,” “colorectal cancer,” “polyps,” “rimonabant,” “cannabidiol,” “cannabinoids,” “azoxymethane,” “xenograft,” and “mice.” Only studies that met the eligibility criteria were included.

**Results:**

Eight *in vivo* experimental studies were included in the analysis after the full-text evaluation. Seven studies were azoxymethane (AOM) colorectal cancer models, and four studies were xenograft models. Cannabidiol botanical substance (CBD BS) and rimonabant achieved high aberrant crypt foci (ACF) reduction (86% and 75.4%, respectively). Cannabigerol, O-1602, and URB-602 demonstrated a high capacity for tumor volume reduction. Induction of apoptosis, interaction with cell survival, growth pathways, and angiogenesis inhibition were the mechanisms extracted from the studies that explain cannabinoids' actions on CRC.

**Conclusions:**

Cannabinoids have incredible potential as antineoplastic agents as experimental models demonstrate that they can reduce tumor volume and ACF formation. It is crucial to conduct more experimental studies to understand the pharmacology of cannabinoids in CRC better.

## 1. Background

Colorectal cancer (CRC) is the third most common cancer worldwide, only behind prostate and lung in males, and behind breast and lung in females [1]. It has high morbidity and mortality that represents a heavy burden for health systems worldwide. In the United States alone, with roughly 1.8 million new cases in 2018, healthcare costs exceed $14 billion annually [2]. In addition, it is the fourth cause of cancer-related deaths [3, 4]. CRC represents a significant public health concern because temporal projections estimate that its global burden will increase by 60% to more than 2.2 million new cases and 1.1 million cancer deaths by 2030 [5].

CRC is a type of cancer with a complex and heterogeneous pathophysiology. It is the result of the transformation of healthy colonic epithelial cells into cancer [6]. This process, called “adenoma-carcinoma sequence,” develops through an ordered series of events, in which the initial step is the transformation of normal colonic epithelium to aberrant crypt foci (ACF) [6]. ACF progress to CRC, in 10–15 years [7]. During this process, many risk factors play an essential role in pathogenesis, including unhealthy diet, smoking, alcohol use, physical inactivity, inflammatory bowel disease, and aging [2].

Breakthroughs in CRC therapy have decreased the mortality of patients with CRC. Current chemotherapeutic options continue to have important side effects due to cytotoxicity and may fail to prevent disease progression [8]. Thus, there is a great interest in new therapeutic approaches for CRC, including phytochemical agents.

Cannabinoids might be substances with possible therapeutic potential for cancer because of their chemotherapeutic effect and their ability to attenuate anorexia, pain, and emesis; these are common side effects of chemotherapy [9, 10]. This has been proved in several experimental models of CRC, brain cancer, breast cancer, lung cancer, prostate cancer, leukemia, and melanoma [11]. However, to the best of our knowledge, cannabinoids have not been tested in humans as medicines for CRC.

Animal models and cell lines of CRC have tested cannabinoids. This study aims to conduct a systematic review of the research about the effect of cannabinoids on *in vivo* azoxymethane (AOM) or xenograft CRC models. The outcomes used to assess the effects of cannabinoids, compared with no cannabinoid therapy, were a decrease in the number of preneoplastic lesions (aberrant crypt foci), number, and volume of neoplastic lesions.

## 2. Materials and Methods

The protocol for this study was registered in PROSPERO (International Prospective Register for Systematic Reviews) under CRD42019148356 [12]. This systematic review was performed following the Preferred Reporting Items for Systematic Reviews and Meta-Analyses (PRISMA) statement (Supplementary file) [13].

### 2.1. Eligibility Criteria

*Population.* The population should be animal species (no restrictions), used for *in vivo* models of CRC, either chemically induced (Azoxymethane or DSS) or by xenograft injection. Dose and time of exposure to azoxymethane were not exclusion criteria for this review. We excluded all studies that included only *in vitro* assessment and studies that evaluated species for noncolorectal cancer models.

*Intervention.* Studies had to evaluate the beneficial effects of the following cannabinoids: CBD, CBG, O-1602, LYR-8, WIN 55, 212–2, AEA, HU-210, rimonabant, anandamide reuptake inhibitors (VDM11), FAAH inhibitors, and MAGL inhibitors.

*Comparators.* Studies had to include at least one comparator group of the same animal species used for the intervention group, with similar characteristics (weight, age, sex, exposure to the same environment, and feeding), without exposition to cannabinoid therapy.

*Studies.* Studies should be experimental *in vivo* studies of CRC in mice, with at least one control group. We excluded conference abstracts, narrative reviews, and systematic reviews.

*Primary Outcome.* There should be a reduction in tumor volume (mm^3^), number of aberrant crypt foci (ACF), and number of tumors comparing intervention and control group.

*Secondary Outcome.* There should be an expression of apoptosis markers (Bax, caspase-3, caspase-9, annexin V, PI), expression of proinflammatory markers (STAT3, NF*κβ*, TNF-*α*), and levels of endocannabinoids.

### 2.2. Search Strategy

We performed a methodologic and systematic strategic search in the following electronic bibliographic databases: PubMed, Scopus, and Embase (from their inception to December 18, 2019). The last search was run on December 18, 2019. Only full available articles written in English were suitable for assessment. We used the following Medical Subject Headings (MESH) terms in PubMed: “colorectal neoplasms”, “colonic neoplasms”, “colorectal cancer”, “polyps”, “rimonabant”, “cannabidiol”, “cannabinoids”, “azoxymethane”, “xenograft”, and “mice”.

### 2.3. Study Selection and Data Collection

The authors EOG and LLT conducted the search independently. Duplicate articles were moved to a different folder and registered in the flowchart. Before the selection process, a test was conducted to evaluate the agreement between evaluators. All titles and summaries of the articles were assessed by EOG and LLT independently based on a selection criterion. The full text of previously selected studies was then reviewed and analyzed. Any disagreement was discussed, and if not resolved, a third author (AVV) was consulted. All selected articles were summarized in a flowchart according to the PRISMA protocol. We used a standardized form with a pilot test to collect the following data: title, author, publication year, type of animal model, sample size, type of cannabinoid, the dose of cannabinoid, the dose of AOM, type of outcome measure, length of the experiment, reduction in ACF formation, reduction in the number of tumors, tumor volume reduction, pathway or function modified by cannabinoids, an increase of endocannabinoid levels, expression of apoptosis markers, and expression of proinflammatory markers.

### 2.4. Quality Assessment

The risk of bias was independently evaluated by two authors (EOG and LLT) following the Systematic Review Centre for Laboratory animal Experimentation (SYRCLE) risk of bias tool [14]. The domains considered were random sequence generation, baseline characteristics, allocation concealment, random housing, blinding, random outcome assessment, outcome assessor blinding, incomplete outcome data, selective outcome report, and other sources of bias (contamination, the influence of funders, and analysis of errors) [14]. We reviewed each article, and we sought if any of these biases were present. Any discrepancy was discussed between 2 authors (EOG and LLT), and if not resolved, a third investigator intervened (AVV).

### 2.5. Data Analysis

Proportions were used as descriptive statistics for primary outcomes. Secondary outcomes were described qualitatively. A meta-analysis or measures of consistency were not performed due to characteristics of the studies and heterogeneity of articles.

## 3. Cannabinoids: Pharmacology and Generalities

Endocannabinoids are lipid mediators, including amides, esters, and ethers of polyunsaturated fatty acids, which were isolated from the porcine brain [15–17]. Anandamide's structure resembles Δ9-THC structure, and it is synthesized from membrane phospholipids by the enzymes N-acyl phosphatidylethanolamine phospholipase *D* (NAPE-D) and lysophospholipase *D* (lyso-PLD) [18]. 2-Arachidonoyl-glycerol (2-AG) is an arachidonoyl ester, produced from diacylglycerols [18]. Endocannabinoids diffuse into the extracellular space and bind to CB1 and CB2 receptors, TRPV1, TRPM8, and GPR55 [18]. Anandamide and 2-AG are reuptake via an extraneuronal monoamine membrane transporter (EMT); then, they are degraded by the fatty acid amide hydrolase (FAAH) and the monoacylglycerol lipase (MAGL), respectively [16, 19]. Most known plant-derived cannabinoids include tetrahydrocannabinol (THC) and cannabidiol (CBD) [20]. These are tricyclic terpenoid compounds bearing a benzopyran moiety soluble in lipids and nonpolar organic solvents [20, 21].

Δ9-Tetrahydrocannabinol (THC) and anandamide have the highest affinity for the CB1 receptor, while CBD exhibits low affinity for these receptors [20, 21]. However, CBD has been proved to enhance endocannabinoid levels and indirectly activate CB receptors [8].

CB1 receptors constitute one of the most abundant receptors in the central nervous system. In the case of CB2 receptors, these are expressed in cells of the immune and hematopoietic system, spleen, and tonsils, modulating cytokine release and cellular immune migration [16]. Both receptors are metabotropic and belong to the *G* protein-coupled receptor (GPCR) family, and their activation produces inhibition of the adenylyl cyclase via *G* proteins (Gi/*o*) [16]. This decreases cAMP in the cell and activity of protein kinase A [16].

Cannabinoids may have alternative molecular targets other than classical CB1 and CB2 receptors [22]. Recently, orphan GPCRs like the GPR 55, GPR18, and GPR110 have been identified as new targets [22]. There is also increasing evidence that they can interact with ionotropic receptors such as the transient receptor potential cation channel subfamily V member 1 (TRPV1), and the transient receptor potential cation channel subfamily *M* member 8 (TRPM8) [23].

The transient receptor potential vanilloid receptor 1 (TRPV1) and the transient receptor potential cation channel subfamily *M* member 8 (TRPM8) are ionotropic channels that allow Na^+^ and Ca^++^ entry to the cell [24]. Cannabidiol (CBD) and cannabigerol (CBG) close the TRPM8 channel, whereas CBD opens TRPV1 [24].

GPR55 is another GPCR, which is coupled to a G*α*12/13 protein [25]. Several cancer lines like OVACAR3 (ovarian cancer cell line), PC-3, and DU145 (prostate cancer cell lines) exhibit expression of this orphan receptor [25]. Furthermore, Piñeiro et al. showed an autocrine activation of this receptor through his main endogenous agonist lysophosphatidylinositol (LPI) [25]. The receptor acts via activation of G*α*12 and Gq family proteins, which activate Ras homolog gene family, member A (RhoA) kinase [26]. Overexpression of GPR55 produces increased levels of pERK in HEK-293, breast carcinoma, and glioma cells, while pAKT levels are increased in ovarian and prostate cancer cells [25, 27].

The high expression of GPR55 is also linked to high proliferation indices in human breast tumors and Glioblastoma [26]. The best-studied cannabinoid with actions on the GPR55 in colonic tissue is O-1602 [28]. It is highly speculative that the compound exerts its antineoplastic effects on CRC tissue through the GPR55 receptor, as the cannabinoid has shown agonist activity on this receptor [28]. More research is needed before we can conclude the actions of cannabinoids on this receptor.

Ceramide's synthesis begins with the enzyme serine palmitoyltransferase (SPT) [29]. Gustaffson et al. have demonstrated that the cannabinoids Win55,212-2 and *R* (+)-methanandamide induce ceramide accumulation mainly through CB1 and CB2 activation, which acts on SPT [30, 31]. Both studies were performed in mantle cell lymphoma cells (L718, L1547, L1676, and Rec-1) [30, 31]. Furthermore, in neural tissues (rat glioma C6 line and H4 neuroglioma), *R* (+)-methanandamide and JWH-133 (CB2 agonist) also induce ceramide accumulation [32, 33]. Ceramide provokes a loss of mitochondrial membrane potential and caspase activation, subsequently [30, 31].

### 3.1. Reported Effects of Cannabinoids on CRC

**Figure 1:**
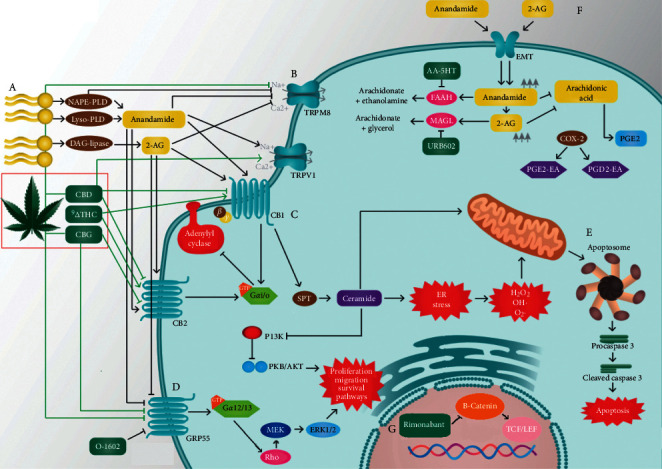
Overview of cannabinoids' pharmacology. (A) Synthesis of endocannabinoids from membrane lipids. (B) TRPM8 and TRPV1, ionotropic receptors of cannabinoids. (C) Cannabinoid (CB) receptors 1 and 2 coupled to g protein and adenylyl cyclase, cannabinoids; their respective ligands are shown in the image. (D) GPR55 receptor coupled to g protein, Rho GTPase, and MAPK pathway; its respective ligands are shown in the image. (E) Ceramide synthesis *de novo* induced by endocannabinoids. (F) Reuptake of endocannabinoids and subsequent metabolism. (G) Apoptosome assembly during the mitochondrial pathway of apoptosis. Executioner caspase is cleaved, and the cell undergoes apoptosis. (H) Rimonabant increases *β*-catenin breakdown and decreases the activity of the T-cell factor/lymphoid enhancer-binding factor (TCF/LEF). Other studied effects of cannabinoids not included in the figure are cytotoxic effects (antiproliferative effects), upregulation of estrogen receptors, reduction of proinflammatory markers, antiangiogenic effects (inhibition of proangiogenic factors), induction of chromosomal abnormalities, and mitotic catastrophe.

Most cultured colonic cancer cells used for *in vitro* assessment express CB1, CB2, TRPM8, and GPR55 (G protein-coupled receptor) [34–39]. Additionally, adenomatous polyps and colorectal cancer tissue have increased the amounts of the endogenous cannabinoids AEA and 2-AG (3-fold versus 2-fold, respectively) [40]. This has been suggested to be a mechanism of self-protection against further tumor progression [40, 41]. Cannabinoids and phytocannabinoids have, therefore, effects on colonic cancer tissues since CRC tissues produce those (endogenous cannabinoids) and express some of their receptors (Figure 1).

One of the main effects demonstrated in experimental models is apoptosis. This is proposed to be mediated through the upregulation of endoplasmic reticulum stress-related genes (ATF-4, TRB3), accumulation of reactive oxygen species (ROS), cell cycle arrest in a p-53 independent manner, and activation of proapoptotic proteins (BAX, caspase 3/9) [38, 39, 42–44]. Ceramide enhanced production is another mechanism of apoptosis induction by cannabinoids through the mitochondrial pathway [45]. In these cases, proapoptotic proteins, usually sequestered in the intermembrane space, are released into the cytosol, assembling the “apoptosome” (formed by the binding of cytochrome c, Apaf-1, and procaspase-9) [46]. Procaspase 3 is cleaved by the apoptosome and causes morphological and biochemical changes seen in apoptosis [46].

Cannabinoids also regulate different proliferation, growth, and survival pathways [45]. Different carcinoma cell lines treated with ∆9-THC also exhibited inhibition of the RAS-MAPK pathway and the phosphatidylinositol 3-kinase pathway (PI3k-AKT) pathway through CB1-receptor activation or ceramide accumulation [34, 45].

*In vitro* studies have also reported that COX-2 metabolites of anandamide (PGE2-EA and PGD2-EA) have growth inhibitory effects in CRC [47–49]. In the case of rimonabant, this is an inverse agonist of CB1 receptors; however, it was demonstrated that this compound counteracts the Wnt/*β*-catenin pathway by decreasing the activity of transcription factors T-cell factor/lymphoid enhancer-binding factor (TCF/LEF) [50, 51]. Other effects of cannabinoids are reported less frequently, including cytotoxic effects similar to 5-Fluorouracil and upregulation of estrogen receptors [52–54].

Contrarily to these findings, in some studies, CB receptors have been involved in CRC origin. CB1 and CB2 receptors were prognostic markers of survival in advanced CRC stages (IV) [36]. Moreover, Martínez et al. demonstrated a biphasic effect of synthetic cannabinoids on colon cancer-derived cell line HT29 [55]. Sub-micromolar concentrations (1 µm) of CB_2_-specific agonists stimulate the PI3K/AKT pathway and the transcription factor SNAIL, promoting cell proliferation in this *in vitro* model [55]. Figure 1 summarizes some of the main mechanisms of cannabinoids on CRC, described in the literature.

## 4. Results

**Figure 2:**
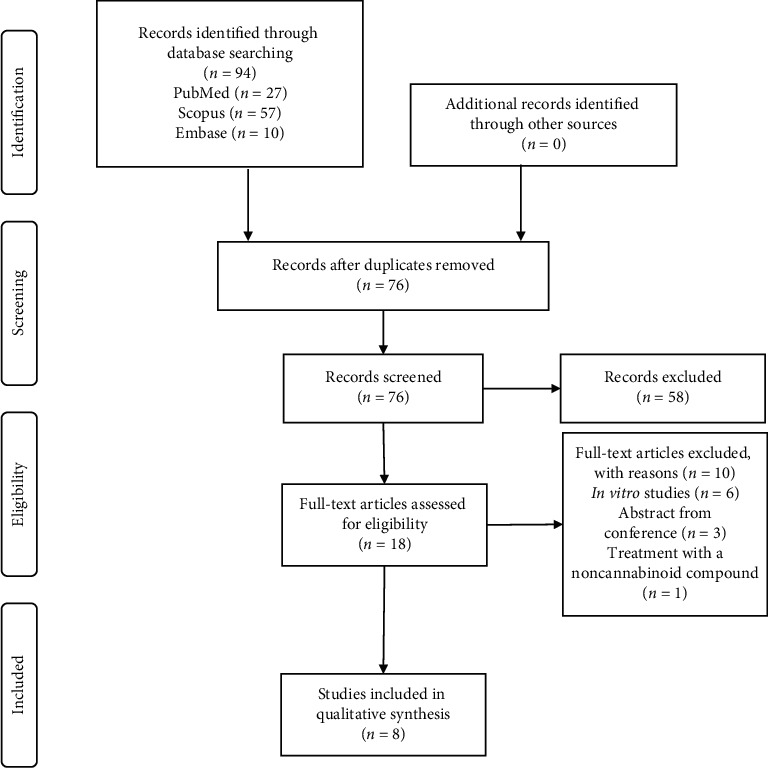
Preferred Reporting Items for Systematic Reviews and Meta-Analysis flowchart detailing the search strategy and study selection output.

We identified 94 records in electronic databases: 57 in Scopus, 27 in PubMed, and 10 in Embase. Duplicate records were removed, and 76 studies remained. After the title and abstract review, 18 studies were selected, of which only 8 were included in the analysis after the full-text evaluation (Figure 2).

Six studies were excluded because they only included *in vitro* assessments, three were abstracts from a conference, and their full-text article could not be found, and one study assessed a noncannabinoid compound.

### 4.1. Characteristics of Included Studies

The characteristics of the included studies are summarized in Table 1. Seven studies were AOM-colorectal cancer models [7, 8, 28, 35, 38, 56, 57]. The protocol for 4 of the studies with this model was four single doses of 10 mg/kg (40 mg/kg in total, intraperitoneally) at the beginning of the first, second, third, and fourth week [7, 8, 38, 56]. Two studies performed a model, in which 3 mg/kg was given intraperitoneally at days 1 and 5 during the first week, 3 mg/kg at days 1 and 5 during the third week, and 2 mg/kg at days 1 and 5 during the 17^th^ week [35, 57]. In Kargl et al.'s research, animals received a single intraperitoneal injection of AOM (10 mg/kg) [28].

One of these studies was a combined model of AOM/DSS (Dextran sodium sulfate) [28]. Four studies were xenograft models of colorectal cancer, which implanted a single cell suspension of either HCT116 (3 studies) [8, 38, 56] or HT29 (1 study) [58] colorectal cancer cells within a heterotopic flank of the host (4 studies) [8, 38, 56, 58]. The most common host used for studies was IRC mice (4 studies) [7, 8, 38, 56], followed by C57BL/6N (2 studies) [35, 57], CD1 mice (1 study) [28], and BALB/c mice (1 study) [58]. Cannabinoids and doses assessed were highly variable across the studies (Table 1).

However, CBD was the most common, being evaluated in two of the studies [7, 8]. Romano et al. tested different c*annabis* extracts, with a high content of CBD [8].

### 4.2. Risk of Bias

Measures to reduce performance bias were not reported in any of the publications, making them highly likely to have performance bias [7, 8, 28, 35, 38, 56–58]. There was a high risk of detection bias in all studies because there was no random outcome assessment [7, 8, 28, 35, 38, 56–58]. We found that other potential sources of bias were present in half of the studies as pharmaceutical companies funded them [7, 8, 38, 57]. Some of the domains had to be scored as unclear risk of bias, as there were few details about the methods used by each author [7, 8, 28, 35, 38, 56–58] (Table 2).

### 4.3. Reported Outcomes

The reduction of ACF formation could be extracted in four of the studies included [7, 8, 35, 57]. CBD BS and Rimonabant achieved the highest reduction (86% and 75.4%, respectively), while sole CBD did not have high reduction percentages (33%) [7, 8, 35, 57]. Only the study of Romano et al. reported a reduction in polyps' formation (79%) with CBD BS [8]. A reduction in the number of tumors was assessed in only three studies, with low reduction percentages [7, 8, 28]. Reduction of volume tumor was more evident in the xenograft models of Kargl et al. and Pagano et al., which used O-1602 and one MAGL inhibitor, respectively [28, 56]. LYR-8 and CBD BDS did not show a marked reduction of volume in the xenografts than the other cannabinoids [8, 58]. Among the mechanisms reported for cannabinoids effects, induction of apoptosis (4 studies) [7, 28, 35, 38], antiproliferative effects (3 studies) [7, 8, 38], and angiogenesis inhibition (2 studies) [56, 58] were the most common (Table 3).

In these studies, specific markers of apoptosis, mainly caspase-3, and caspase-9, were augmented in CRC tissues treated with CBD, CBG, AA-5HT, VDM11, HU210, and O-1602 [7, 28, 35]. Caspase enzymes are responsible for the protease cascade of apoptosis, being hallmarks of programmed cell death [46].

Borrelli et al. demonstrated that CBG antagonizes TRPM8 through inhibition of intracellular calcium increase, with a rise in apoptosis markers [38]. Pharmacological enhancement of endocannabinoid levels was observed in three of the included studies [7, 35, 56]. Izzo et al. used in their study AA-5HT, a FAAH inhibitor, and VDM11, an endocannabinoid reuptake inhibitor, while Pagano et al. assessed URB602, a MAGL inhibitor [35, 56]. On the other hand, Romano et al. demonstrated an antiproliferative effect of CBD in MTT assay, by indirect activation of CB1 and CB2 receptors [8].

Two studies demonstrated that angiogenic factors (VEGF, FGF, and HIF) were downregulated by the direct action of URB602 and the synthetic cannabinoid LYR-8 [56, 58]. In the case of rimonabant, it provoked a phenomenon called “mitotic catastrophe” as its mechanism of action [50]. Mitotic catastrophe is a phenomenon that has been studied in MCF7 and HeLa cells, in which cell cycle progression is inhibited due to an arrest of cells in the G2/M phase of the cell cycle [59]. This is provoked by high amounts of damaged DNA (including high amounts of cells with polyploidy) and apoptosis induction through activation of p53 [59]. This molecular effect is characteristic of the antineoplastic medicine paclitaxel [59]. Other reported effects of cannabinoids include the reduction of proinflammatory markers [7, 28]. Kargl et al. showed that SW480 and HT-29 cells treated with O-1602 could reduce the expression of TNF-*α*, phosphorylation of NF*κβ*, and STAT3. TNF-*α* is a cytokine, which exerts a dual role in immunomodulation (inflammation, immune surveillance, and hematopoiesis) and tumorigenesis [60], while the transcription factor, NF*κβ*, regulates proinflammatory cytokines (TNF-*α* and IL-1), and it has a pathogenic function in cancer and inflammation [61, 62]. In the case of STAT3, this belongs to a family of proteins, activated mainly by IL-6, which are linked to inflammation-associated tumorigenesis [63].

## 5. Discussion

It was observed across the studies that cannabinoids can reduce the development of preneoplastic lesions (ACF) and tumor growth of colorectal cancer in chemically induced CRC and xenograft models. ACF arise from a stem cell in the colonic crypts and represent abnormalities before polyp formation, being regarded as the earliest preneoplastic lesions of CRC [64]. The most common genetic changes involved in ACF formation are mutations of the protein KRAS and microsatellite instability; both are key events in the two main pathways of CRC genesis [64]. Cannabinoids (CBD, AA-5HT, VDM11, HU210, and rimonabant) have chemopreventive potential, as they were able to attenuate colon carcinogenesis *in vivo* [7, 8, 35, 57].

The MTT (3-[4,5-dimethylthiazol-2-yl]-2,5 diphenyl tetrazolium bromide) assay determines the mitochondrial activity an indirect measure of the number of viable cells [65]. CBD and CBG have cytotoxic effects on CRC tissue and cells, as they were tested either by MTT assay or ^3^H-thymidine incorporation with a significant decrease of cell proliferation [7, 8, 38]. This makes them possible candidates for further research on CRC cytotoxic drugs.

In a prospective human study, researchers achieved a tumor volume reduction rate (measured by MRI-volumetric techniques), of 51.7% in patients treated with four cycles of FOLFOX [66]. In this systematic review, we found that various cannabinoids could achieve similar reduction rates of the tumor volume (33.18%-52%, for *in vivo* research).

All studies used a diverse variety of cannabinoid compounds and doses, and several physiological mechanisms were elucidated [7, 8, 28, 35, 38, 56–58]. Among them, we highlight apoptosis, angiogenesis inhibition, and mitotic catastrophe [7, 8, 28, 35, 38, 56–58]. Half of the studies exhibited an increase in expression of executioner caspases, demonstrating that CBD, CBG, AA-5HT, VDM11, HU210, and O-1602 have apoptosis as their main mechanism against CRC [7, 28, 35, 38].

Tumor growth and metastasis depend on angiogenesis because, in the absence of vascular support, tumors may become necrotic or even apoptotic [67]. URB-602 and LYR-8 have antiangiogenic mechanisms, as proven in their studies, with similar actions to bevacizumab (inhibiting VEGF) [56, 58].

The strengths of this review include being the first study to our knowledge that has systematically reviewed the experimental evidence of cannabinoids in colorectal cancer, as there is no study in humans to date. The review used a comprehensive search strategy in a wide range of registries and data sources and used a well-known quality assessment (SYRCLE's risk bias tool) [14], and the manuscript was registered in the PROSPERO [12]. However, some limitations were found. The main limitation of this study was that animal models, outcomes, and cannabinoids tested were not the same across studies [7, 8, 28, 35, 38, 56, 57]. The quality assessment indicated that all the studies had methodological limitations and were at risk of bias [7, 8, 28, 35, 38, 56,57]. The eight studies did not explicitly state blinding and allocation concealment on their methods [7, 8, 28, 35, 38, 56, 57]. Thus, it was impossible to determine if certain risks were present. Four studies were founded by pharmaceutical companies, which represent a significant bias [7, 8, 38, 57]. We believe that these were not significant drawbacks, as we did not perform a meta-analysis.

Current chemotherapy generates high costs and high toxicity for CRC treatment [68]. CRC therapy includes regimes such as FOLFOX (5-fluorouracil, leucovorin, and oxaliplatin), FOLFIRI (5-fluorouracil, leucovorin, and irinotecan), recombinant monoclonal antibodies like cetuximab, and bevacizumab [68]. It has been reported that FOLFOX and FOLFIRI have a high incidence of neutropenia (41.7% and 24%, respectively); additionally, a high percentage of patients (18%) under FOLFOX develop neurologic symptoms due to toxicity, and in a high percentage of patients treated with cetuximab, gastrointestinal symptoms like diarrhea are present (13-25%) [69]. Moreover, chemotherapy during eight weeks in the USA for CRC is estimated to generate high costs for FOLFOX/bevacizumab ($21033) and FOLFIRI/cetuximab ($30675) [70].

New molecules as effective as the current chemotherapy need to be synthesized, with less cytotoxic effects. Cannabinoid molecules are not as expensive as monoclonal antibodies and are proven that they have antiproliferative, proapoptotic, and chemopreventive effects on CRC. The next step is to conduct more *in vivo* research of these compounds, before proceeding to human beings, to determine whether these results are reproducible.

## 6. Conclusions

Overall, there is no robust evidence so far to introduce cannabinoids to the clinical practice as adjuvants of chemotherapy or the main treatment of CRC because no human models have tested these compounds, and only eight *in vivo* experimental models have been conducted and reported in the literature, during the assessed period. Nevertheless, current literature findings demonstrate that cannabinoids might have potential as antineoplastic agents because they can reduce tumor volume and ACF formation. Induction of apoptosis through several mechanisms is the main action of cannabinoids on CRC, while inhibition of angiogenesis and mitotic catastrophe were also reported. It is crucial to conduct more experimental studies before conducting research on humans, providing high-quality evidence on the efficacy and safety of cannabinoids for treating CRC.

## Figures and Tables

**Table 1:** Summary of study characteristics.

Author, year	Animal model	Sample size and number of groups in the AOM model	Sample size and number of groups in the xenograft model	Cannabinoid and dose^¶^	Main outcome measures	Length of the experiment
Aviello, et al. 2012 [7]	ICR male mice AOM-induced CRC	*N* = 10 per group. Group 1 = Vehicle Group 2 = AOM plus vehicle Group 3 = AOM plus CBD Group 4 = AOM plus CBD	N/A	Experimental group 3: CBD 1 mg/kg IP Experimental group 4: CBD 5 mg/kg IP Three times per week	1. Decrease in ACF formation 2. Increase in expression of cyclooxygenase-2 (COX-2), inducible nitric oxide synthase (iNOS), and caspase-3 3. Increase in phosphorylation of AKT 4. MTT assay for the antiproliferative effect 5. Increase in endocannabinoids levels	Three months

Borrelli, et al. 2014 [38]	ICR male and female mice AOM-induced CRC/Xenograft model of HCT 116 colon carcinoma cells	*N* = 10 per group. Group 1 = Vehicle Group 2 = AOM plus vehicle Group 3 = AOM plus CBG Group 4 = AOM plus CBG	HCT 116 cells (2.5 × 10^6^) were injected subcutaneously into the right flank of each athymic mice. At 10 days after inoculation (once tumors had reached a size of 550–650 mm^3^), mice were randomly assigned to one control group and three treated groups	AOM model: Experimental group 3: CBG 1 mg/kg IP Experimental group 4: CBG 5 mg/kg IP three times per week Xenograft model: Experimental group 1: CBG 1 mg/kg IP Experimental group 3: CBG 3 mg/kg IP Experimental group 4: CBG 10 mg/kg IP t hree times per week	1. Decrease in tumor growth 2. Decrease in ACF formation 3. Decrease in CB receptors expression 4. MTT assay for antiproliferative effect 5. CBG antagonism at TRPM8 receptor 6. Increase in ROS production 7. Increase in CHOP mRNA expression 8. Increase in caspase 3/7 enzymatic assay	Three months

Izzo, et al. 2008 [35]	C57BL/6N female mice AOM-induced CRC	*N* = 6 per group. Group 1 = vehicles Group 2 = AOM plus the vehicle Group 3 = AOM plus AA-5HT Group 4 = AOM plus VDM11 Group 5 = AOM plus HU210	N/A	AA-5HT^*∗*^: 5 mg/kg IP N-(2-methyl-3-hydroxy-phenyl)-5,8,11,14-eicosa-tetraenamide]) ^*∗∗*^: 5 mg/kg IP HU210 : 0.1 mg/kg IP Given daily during the experiment	1. Decrease in ACF formation 2. Increase in endocannabinoids levels 3. Increased expression of caspase-3 and caspase-9	Six months

Kargl, et al. 2013 [28]	CD1 male mice AOM/DSS-induced CRC	*N* = 12 per group Group 2 = AOM/DSS plus the vehicle Group 1 = AOM/DSS plus O-1602	N/A	O-1602 3 mg/kg IP every second day over four weeks	1. Decrease in tumor growth 2. Decrease in phosphorylation of NF*κβ* and STAT3 3. Increase in expression of BAX and P534 . Increase in a nnexin V/PI expression 5. Decreased expression of TNF-*α*	Twelve weeks

Pagano, et al. 2017 [56]	ICR male or female mice AOM-induced CRC/Xenograft model of HCT 116 colon carcinoma cells (*N* = 10)	*N* = 10 per group. Group 1 = Vehicles Group 2 = AOM plus vehicle Group 3 = AOM plus URB-602	HCT 116 cells (2.5 × 10^6^) were injected subcutaneously into the right flank of each athymic mice and at 10 days after inoculation (once tumors had reached a size of 250–300 mm^3^), mice were randomly assigned to one control and treated group. *N* = 5 animals per group	AOM and xenograft model:URB-602 ^†^ 5 mg/kg IP three times a week	1. Increase in expression of monoacylglycerol lipase (MAGL) in CRC cells and xenograft tissue 2. Decrease in tumor growth 3. Increase in endocannabinoid levels 4. Decreased expression of VEGF and FGF-2 5. Decrease in cyclin-D1 and p27KIP expression	Three months

Romano, et al. 2014 [8]	ICR male mice AOM-induced CRC/Xenograft model of HCT 116 colon carcinoma cells	^††^ Group 1 = Vehicle Group 2 = AOM plus vehicle Group 3 = AOM plus CBD BDS	HCT 116 cells (2.5 × 10^6^) were injected subcutaneously into the right flank of each athymic mice and at 10 days after inoculation (once tumors had reached a size of 300 mm^3^), mice were randomly assigned to control and treated group with CBD BDS	AOM and xenograft model: CBD BDS 5 mg/kg IP three times a week	1. Decrease in tumor growth 2. Decrease in ACF formation 3. MTT assay for the antiproliferative effect	Three months

Santoro, et al. 2009 [57]	C57BL/6N female mice AOM-induced CRC	*N* = 9 per group. Group 1 = Vehicle Group 2 = AOM plus vehicle Group 3 = AOM plus rimonabant	N/A	Rimonabant 3 mg/kg IP given daily during the experiment	1. Decrease in ACF formation 2. Increase in expression of cyclin B1 /cdk1 complex 3. Decreased phosphorylation of p38/MAPK and PARP-1 4. Increase in mitotic index, polyploidy, and chromosome aberrations 5. Decreased phosphorylation of Chk1	Six months

Thapa, et al. 2012 [58]	BALB/*c* nude mice xenograft model of HT-29 of colon carcinoma cells	N/A	HT 29 cells (5 × 10^6^) were injected subcutaneously into the rear flanks of each BALB/*c* nude mice and once tumors had reached a size of 50 mm^3^, mice were randomly assigned to control and treated groups. *n* = 6 animal per group	LYR-8 10 mg/kg IP given daily during the experiment	1. Decrease in tumor growth 2. Decreased expression of COX-2, vascular endothelial growth factor (VEGF), and hypoxia-inducible factor (HIF-1*α*)	Unknown

^*∗*^FAAH inhibitor. ^*∗∗*^VDM11 inhibitor. ^†^MAGL inhibitor. ^¶^All doses were started one week before the first injection of AOM. ^††^Sample size is not described in the article. CBD: cannabidiol, CBG: cannabigerol, IP: intraperitoneal, AOM: azoxymethane, AA-5HT: N-arachidonoyl-serotonin, BDS: botanical drug substance, ACF: aberrant crypt foci, ROS: reactive oxygen species, N/A: not applicable.

**Table 2:** Quality assessment.

Author	Selection bias			Performance bias		Detection bias		Attrition bias	Reporting bias	Other biases
	Random sequence generation	Baseline characteristics	Allocation concealment	Random housing	Blinding	Random outcome assessment	Outcome assessor blinding	Incomplete outcome data	Selective outcome report	Other sources of bias
Aviello, et al. 2012 [7]	Unclear	Yes	Unclear	Yes	Unclear	No	Unclear	Yes	Yes	Yes
Borrelli, et al. 2014 [38]	Unclear	Yes	Unclear	Yes	Unclear	No	Unclear	Yes	Yes	Yes
Izzo, et al. 2008 [35]	Unclear	Yes	Unclear	Yes	Unclear	No	Unclear	Yes	Yes	No
Kargl, et al. 2013[[28]	Unclear	Yes	Unclear	Yes	Unclear	No	Unclear	Yes	Yes	Yes
Pagano, et al. 2017 [56]]	Unclear	Yes	Unclear	Yes	Unclear	No	Unclear	Yes	Yes	No
Romano, et al. 2014 [8]	Unclear	Yes	Unclear	Yes	Unclear	No	Unclear	Yes	Yes	No
Santoro, et al. 2009 [57]	Unclear	Yes	Unclear	Yes	Unclear	No	Unclear	Yes	Yes	Yes
Thapa, et al. 2012 [58]	Unclear	Yes	Unclear	Yes	Unclear	No	Unclear	Yes	Yes	No

SYRCLE's risk of bias tool for animal studies [14].

**Table 3:** Summary of main outcomes.

Author, year	Decrease in ACF formation	Decrease in the number of tumors	Mean tumor volume		Decrease in tumor volume	Main reaction, pathway or function modified by cannabinoids	Increase in endocannabinoid levels	Increase in expression of apoptosis markers	Changes in expression of proinflammatory markers
			Control	Experimental					
Aviello, et al. 2012 [7]	CBD: 1 mg/kg 33.3% CBD: 5 mg/kg 33.3%	CBD: 1 mg/kg 55.5% CBD: 5 mg/kg 22.2%	N/A	N/A	N/A	Apoptosis, antiproliferative effect	2-AG Anandamide	Increased expression of caspase 3	No significant changes in cyclooxygenase 2 (COX-2) and inducible oxide nitric synthase (iNOS) expression

Borrelli, et al. 2014 [38]	N/A	N/A	(2500 ± 414 mm^3^)	(1367 ± 243 mm^3^)	45.3%	Apoptosis, antiproliferative effect	N/A	Increased expression of caspase 3 Increased expression of caspase 7	Increased production of reactive oxygen species (ROS) only in CRC cells (Caco-2 cells)

Izzo, et al. 2008 [35]	AA-5HT: 50.8% VDM11 : 37.5%HU210 : 60.2%	N/A	N/A	N/A	N/A	Apoptosis	2-AG Anandamide	Increased expression of caspase 3	N/A

Kargl, et al. 2013 [28]	N/A	30%	N/A	N/A	50%	Apoptosis	N/A	Increased expression of annexin V/PI	^*∗*^Decreased phosphorylation of NF*κβ* ^*∗∗*^Decreased phosphorylation of STAT3 ^†^Decreased expression of TNF-*α*

Pagano, et al. 2017 [56]	N/A	N/A	(1980 ± 269 mm^3^)	(956 ± 180 mm^3^)	52%	Angiogenesis	2-AG	N/A	N/A

Romano, et al. 2014 [8]	86%	40% no statistical significance achieved	(1130 ± 171.6 mm^3^)	(755 ± 124 mm^3^)	33.18%	Antiproliferative effect	N/A	N/A	N/A

Santoro, et al. 2009 [57]	75.4%	N/A	N/A	N/A	N/A	Mitotic catastrophe	N/A	N/A	N/A

Thapa, et al. 2012 [58]	N/A	N/A	(507 ± 51 mm^3^)	(271 ± 33 mm^3^)	46.5%	Angiogenesis	N/A	N/A	N/A

CBD: cannabidiol, N/A: not applicable. ^*∗*^Nuclear factor kappa-light-chain-enhancer of activated B cells. ^*∗∗*^Signal transducer and activator of transcription 3. ^†^Tumor necrosis factor-alpha.

## Data Availability

The datasets used and/or analyzed during the current study are available from the corresponding author upon reasonable request.

## Abbreviations

TRPV1: Transient receptor potential receptor vanilloid 1 receptor
TRPM8: Transient receptor potential cation channel subfamily M member 8
CB: Cannabinoid
GPCR: G protein-coupled receptor
ER: Endoplasmic reticulum
CBD: Cannabidiol
CBG: Cannabigerol
THC: Tetrahydrocannabinol
NAPE-D: N-acyl phosphatidylethanolamine phospholipase D
lyso-PLD: Lysophospholipase D
2-AG: 2-Arachidonoyl-glycerol
EMT: Extraneuronal monoamine membrane transporter
FAAH: Fatty acid amide hydrolase
MAGL: Monoacylglycerol lipase
COX-2: Cyclooxygenase-2
AA-5HT: N-arachidonoyl-serotonin
ROS: Reactive oxygen species
SPT: Serine palmitoyltransferase
PKB: Protein kinase B
TCF/LEF: T-cell factor/lymphoid enhancer-binding factor
CRC: Colorectal cancer
ACF: Aberrant crypt foci
FOLFOX: 5-Fluorouracil, Leucovorin, Oxaliplatin
FOLFIRI: 5-Fluorouracil, Leucovorin, Irinotecan
AOM: Azoxymethane
DSS: Dextran sodium sulfate.

## Supplementary Materials

Supplementary Materials Supplementary file 1: the PRISMA statement of this study. Supplementary file 2: the search strategy used for this study.
